# Country Physicians

**Published:** 1870-10

**Authors:** 


### COUNTRY PHYSICIANS.

An intelligent correspondent writes: "Your profession, as a general fact, in rural localities, are reading so little that the world is losing confidence in them. The public, in short, is moving away from them by the assistance of such intelligence as you and others, and the schools, are imparting."

No physician, old or young, can afford to do without taking at least two medical periodicals—one general and standard, as the *London Lancet*, which records monthly the progress made throughout the civilized world in all that pertains to medicine and surgery. It is republished in this city at five dollars a year. In addition, each physician should take a more local medical magazine, because each locality has its peculiarities to which the practice must be adapted. Losing sight of this is the reason why a remedy for a given disease is efficient in one place, and is inefficient five hundred miles away. Hence, a New Englander should take the *Boston Medical and Surgical Journal*, while the *Medical and Surgical Reporter* of Philadelphia is better adapted to Pennsylvania and southward. The Chicago monthly magazines are better suited to the great Northwest. The true and educated physician should not only take two or three medical publications, but should be a watchful and conscientious contributor of all observations which come to him that are new, true, practical and important.

It sometimes happens that the phase of a disease in one locality this season is different from what it was last season—hence, requires a modification of the usual remedy. These phases sometimes change during the same season. Hence, the live practitioner is always on the alert, and the very moment a favorite remedy for an ailment fails of its accustomed effect, he sets himself about ferreting out the cause, and changes his practice accordingly ; and he who is most wide awake as to these points will uniformly carry off the palm of success in his locality

